# Supplementary material for: Content-rich biological network constructed by mining PubMed abstracts
Source: BMC Bioinformatics. 2004 Oct 8;5:147. doi: 10.1186/1471-2105-5-147 (PMC528731; doi:10.1186/1471-2105-5-147)
Supplement: Additional File 2 — The original results of the above study (non-essential files are deleted to keep the file size under the limit set by BMC bioinformatics). [file 1471-2105-5-147-S2.bz2 › chilibotAdditionalFile2/dip05/701ID2828368E18034/html/CYSN_CYSD.html]

 


 **CYSN** and **CYSD** 
  
Found 1720 abstracts in PubMed, retrieved 05.  
 

 What does Google say? 
 PDF only 
| .edu only 

---

**Interactive relationship** (e.g. stimulation, inhibition, etc)

**Non-interactive relationship** (e.g. studied together, co-existance, homology, etc.)

- One hundred children with suspected congenital and or malformation and their parents who reported to SAT  [ **CYSD** ]   [ **CYSN** ]  hospital, Medical College, Trivandrum, India formed the test group.  Ref: 12884525 Genetika, 2003
- Both hikers started running for their lives when all of a sudden the CEO stopped, sat  [ **CYSD** ]   [ **CYSN** ]  down on a rock and started to put on running shoes.  Ref: 12886657 Mich Health HospMich Health Hosp, 2003
- Two groups were restored with the SAT  [ **CYSD** ]   [ **CYSN** ]  and 2 by etching the ionomer surface sandwich technique ST.  Ref: 12889705 Pediatr DentPediatr Dent, 2003
- Furthermore, students who are in BSP have statistically lower high school grade point averages GPAs and Scholastic Achievement Test SAT  [ **CYSD** ]   [ **CYSN** ]  scores than students not in BSP.  Ref: 12888847 Cell Biol EducCell Biol Educ, 2003
- CONCLUSIONS The SAT  [ **CYSD** ]   [ **CYSN** ]  is a less complex, quicker, and feasible alternative for the bonding of glass ionomer cements to composite resins in primary molars.  Ref: 12889705 Pediatr DentPediatr Dent, 2003
- RESULTS The SAT  [ **CYSD** ]   [ **CYSN** ]  and ST did not show statistically significant differences in both tests.  Ref: 12889705 Pediatr DentPediatr Dent, 2003
- Majority Asian and White students in BSP graduate with statistically similar UC GPAs despite having lower SAT  [ **CYSD** ]   [ **CYSN** ]  scores than non BSP majority students.  Ref: 12888847 Cell Biol EducCell Biol Educ, 2003
- PURPOSE The purpose of this study was to evaluate a new simultaneous activation technique SAT  [ **CYSD** ]   [ **CYSN** ]  for the union between glass ionomer cements and composite resins.  Ref: 12889705 Pediatr DentPediatr Dent, 2003
- Sequencing showed that the class 1 integron bearing strain contained two distinct gene cassettes, sat  [ **CYSD** ]   [ **CYSN** ]  1 and aadA.  Ref: 12890322 Emerg Infect Dis, 2003
